# Influence of Mineral Powder Content and Gradation on the Aging and High-Temperature Rheological Properties of Styrene-Butadiene-Styrene (SBS) Modified Asphalt

**DOI:** 10.3390/ma18122785

**Published:** 2025-06-13

**Authors:** Chengwei Xing, Zhibin Chang, Bohan Zhu, Tian Jin, Qing Ma, Jie Wang

**Affiliations:** 1 Key Laboratory of Transport Industry of Road Structure and Material, Research Institute of Highway, Ministry of Transport, Beijing 100088, China; j.wang@rioh.cn; 2 Key Laboratory for Special Area Highway Engineering, Ministry of Education, Chang’an University, South 2nd Ring Road Middle Section, Xi’an 710064, China; xingcw@chd.edu.cn (C.X.); zhubh@chd.edu.cn (B.Z.); 3 School of Highway, Chang’an University, South 2nd Ring Road Middle Section, Xi’an 710064, China; 4 The Key Laboratory of Road and Traffic Engineering, Ministry of Education, Tongji University, Shanghai 200080, China; jintian@tongji.edu.cn

**Keywords:** SBS modified asphalt, mastic, mineral powder, rheological properties, gradation

## Abstract

This paper aims to explore the influences of the content and gradation of mineral powder on the rheological properties of styrene-butadiene-styrene (SBS) modified asphalt mastic at different aging stages and temperatures. In the experiment, SBS modified asphalt mastic samples with different powder-to-binder ratios (0.6, 0.8, and 1.0) and different mineral powder gradations (500 mesh passing rates of 76.89% and 100%) were prepared. Following aging periods of 5, 25, and 45 h in the pressure aging vessel (PAV), the asphalt underwent comprehensive rheological characterization using a dynamic shear rheometer (DSR). The research shows that mineral powder can boost mastic’s deformation resistance and elastic effect. When aged by PAV for 45 h, the powder-to-binder ratio increased from 0.6 to 1.0, and its complex modulus increased by nearly 2.5 times at 58 °C. For SBS modified asphalt mastic of PAV 0 h, the powder-to-binder ratio increased from 0.6 to 1.0 and its phase angle was reduced from 59.6 to 53.2, which indicated that the elasticity of mastic was improved. However, this accelerated the degradation rate of SBS, making the aging process more complex. Fine-grained mineral powder is more effective in enhancing mastic’s deformation resistance than coarse-grained mineral powder. The fine-graded mastic had better rutting resistance after 45 h of aging than after 25 h of aging because the mineral powder compensated for the SBS loss-induced elasticity reduction. Smaller mineral powder particles lead to better a mastic anti-aging effect. After 45 h of aging, fine-grained mineral powder offered a better elastic effect. But the ways in which mineral powder and SBS boost mastic elasticity differ greatly. The results of this study provide a reference for optimizing the design of asphalt mixtures.

## 1. Introduction

Asphalt mixtures are widely used in road engineering [1,2]. Mastic composed of asphalt and fillers plays the roles of bonding aggregates and filling voids in asphalt mixtures [3,4,5,6,7]. As the primary dispersion system in asphalt mixtures, mastic has a significant impact on the technical properties of asphalt mixtures, which in turn determines the service performance and lifespan of asphalt pavement [8,9,10]. E, et al. [11] pointed out that the interaction between asphalt mastic and aggregates plays an important role in the overall properties of asphalt mixtures and their durability in flexible pavement. In addition, Ma, et al. [12] found that the creep properties of asphalt and its mastic have a significant impact on the high-temperature rutting resistance of asphalt concrete. Excellent properties of asphalt pavement can be achieved through better design of road materials [13,14,15,16]. As an indispensable component of mastic, mineral powder reacts with the acidic components in asphalt, thereby improving the bonding force between mastic and aggregates. Some scholars have already conducted in-depth research on the properties, types, and dosages of mineral powder, aiming to ascertain the specific impacts of mineral powder on the properties of mastic and have recommended the research findings for practical production and application. Wang, et al. [17] found that increasing the specific surface area of mineral powder or reducing the fineness, plasticity index, and SiO_2_ content of mineral powder could improve the high-temperature properties of asphalt mastic, but it also increased mastic’s high-temperature viscosity, thus affecting the construction fluidity. Therefore, a comprehensive consideration is needed. Giustozzi, et al. [18] pointed out that, compared with asphalt, the complex modulus of asphalt mastic increases both at high and low temperatures. Li, et al. [19] found that the strengthening effect of limestone mineral powder on SBS modified asphalt mastic initially increased linearly with increasing dosage of mineral powder and then was no longer affected by the dosage of mineral powder.

In the actual application process, asphalt mastic will age to varying degrees under the influence of traffic loads and natural environmental factors. Some scholars have already carried out research on the aging of asphalt mastic. For example, Movilla-Quesada, et al. [20] reported that cellulose incineration ash helped to reduce the aging sensitivity of asphalt. Liu, et al. [21] showed that the ultraviolet aging resistance of asphalt mastic was superior to that of the corresponding base asphalt. Therefore, the anti-aging properties of asphalt were improved after it was mixed with filler particles. Furthermore, Xing, et al. [6] concluded through their research that the presence of mineral fillers could change the relative aging rates of the base asphalt and the SBS in SBS modified asphalt. According to their mineralogical differences, mineral fillers can be classified as active fillers (such as fly ash and hydrated lime) and inert fillers (such as granite and limestone) [22,23]. Different mineral fillers have different effects on the anti-aging properties of asphalt. Lesueur, et al. [24] found that hydrated lime slowed down the aging of asphalt more effectively than other less active fillers, such as hydraulic lime and Portland cement. Das, et al. [25] reached a similar conclusion.

Although there have been some research achievements on the effects of mineral powder on the properties of asphalt mastic, most of the studies have not taken into account that the asphalt mastic will age to varying degrees during the actual application process. Based on previous studies, this paper subjected SBS modified asphalt mastic with different powder-to-binder ratios to different degrees of aging to simulate the short-term and long-term aging processes that occur in actual applications and analyzed the effects of the powder-to-binder ratio on the properties of aged SBS modified asphalt mastic. Meanwhile, taking limestone mineral powder as the research object, this paper investigated the effects of mineral powder gradation on the properties of SBS modified asphalt mastic. The research findings provide a reference for practical engineering applications to improve the pavement performance of asphalt mixtures.

## 2. Materials and Methods

### 2.1. Raw Materials

The base asphalt used in this study was 90# bitumen, shear-mixed with 4.5% by weight of linear SBS. The basic properties of the asphalt are presented in Table 1.

In this paper, limestone mineral powder was selected as the raw material for preparing SBS modified asphalt mastic. After measurement, the density of the limestone mineral powder was 2.67 g/cm^3^.

### 2.2. Preparation of Mastic Samples

#### 2.2.1. Preparation of Mineral Powder with Different Gradations

With the help of a 0.075 mm sieve and a 500 mesh sieve, the limestone mineral powder that passed through the 0.075 mm sieve was mixed with mineral powder between 500 mesh and 0.075 mm to obtain limestone mineral powder with a 500 mesh passing rate of 76.89%. Mineral powder with a 500 mesh passing rate of 100% was screened out by taking an equal mass of the mineral powder below the 500 mesh sieve.

#### 2.2.2. Preparation of SBS Modified Asphalt Mastic

SBS modified asphalt mastic is formed by dispersing mineral powder in asphalt. During the preparation of mastic, it is necessary to prevent the mineral powder from caking to ensure uniform dispersion of the mineral powder in the asphalt. A sufficiently long stirring time can improve the uniformity of mixing between the asphalt and the mineral powder [24,26]. The mineral powder was added to the SBS modified asphalt in three batches, and then it was stirred evenly using a shearing machine at a shearing rate of 3000 revolutions per minute (rpm) for 30 min. In this paper, limestone mineral powder was selected to prepare limestone mineral powder SBS modified asphalt mastic samples with powder-to-binder ratios of 0.6, 0.8, and 1.0, as well as limestone mineral powder SBS modified asphalt mastic samples with a powder-to-binder ratio of 0.8 and 500 mesh passing rates of 76.89% and 100%, respectively.

#### 2.2.3. Preparation of Aged Samples of SBS Modified Asphalt Mastic

**Figure 1:**
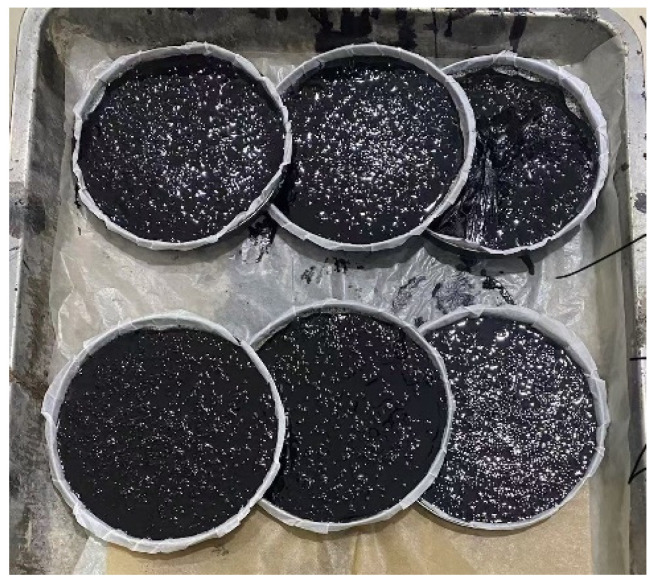
Aged SBS-modified asphalt mastic samples.

In this paper, referring to the improved aging test method for SBS modified asphalt mastic proposed by Xing, et al. [6], both short-term aging and long-term aging followed the pressure aging vessel (PAV) test in accordance with ASTM standard D6521 [27]. The PAV test for 5 h was used to simulate the short-term aging of the SBS asphalt mastic, and on this basis, long-term aging tests for 20 h and 40 h were carried out. Some of the aged samples are shown in Figure 1.

After the aged SBS modified asphalt mastic samples had cooled down, the samples were taken out of the aging trays and the oil paper at the bottom of the samples was torn off. Before conducting other experimental tests, the mastic was re-stirred evenly with a high-speed shearing machine, which ensured that the powder-to-binder ratio of the samples remained unchanged before and after aging. For the convenience of explanation, the abbreviations defining different asphalt mastic samples are listed below (Table 2).

### 2.3. Test Methods

#### 2.3.1. Temperature Sweep Test

The temperature sweep test is a variety of the temperature rheological property tests conducted using a dynamic shear rheometer (DSR). Before the test, a linear viscoelastic range test was first carried out, and a shear strain of 1% and an angular frequency of 10 rad/s were selected. According to ASTM standard D7175 [28], the test rotor was a parallel plate with a diameter of 25 mm and a gap of 1 mm. The test temperature was controlled to be in the range of 58 °C to 70 °C, with a step size of 6 °C. Under these conditions, the complex modulus (G*) and phase angle (δ) of the asphalt at different temperatures were measured.

#### 2.3.2. Multiple Stress Creep and Recovery Test

The multiple stress creep and recovery test (MSCR) is a test for evaluating the high-temperature rutting resistance of asphalt mastic using a DSR. The tests were carried out at 58 °C, 64 °C, 70 °C, and 76 °C, respectively, which conformed to the AASHTO T350 [29] standard. Each sample underwent a cycle consisting of 1 s loading and 9 s recovery, and a total of 30 cycles were conducted. The load for cycles 11 to 20 was 0.1 kPa, and the load for the last 10 cycles was 3.2 kPa. The test collected data from the 10th second to the 30th second. The first 10 s were used for adjusting the sample. Two key evaluation parameters could be calculated from the data obtained in the MSCR test, namely, the recovery rate (R) and the irrecoverable creep flexibility (Jnr).

#### 2.3.3. Frequency Sweep Test

The test temperature of the frequency sweep test was 55–75 °C, with a step size of 10 °C. During the test, a 25 mm parallel plate was used, the shear strain was 1%, and the frequency was 0.1–100 rad/s. The frequency sweep data were processed according to the time–temperature equivalence principle, and the master curves of the complex modulus and phase angle were plotted. Currently, the main models commonly used to construct master curves in asphalt rheology include the generalized Maxwell model, the 2S2P1D model, and so on [30,31]. Since the generalized logistic sigmoidal model has fewer mathematical parameters and a better fitting correlation, this model is more suitable for use in the master curve of the complex modulus [32].

The displacement factor α(T) can be calculated according to the WLF formula, as follows:

(1) $$\mathrm{lg}\;\alpha \left(T\right)=\frac{-C_{1}\left(T-T_{r}\right)}{C_{2}+T-T_{r}}$$

In the formula, T and $T_{r}$ are the test temperature and the reference temperature, respectively, in degrees °C; $C_{1}$ and $C_{2}$ are material constants that depend on $T_{r}$.

The sigmoidal equation was selected to fit the master curve of the complex modulus, as shown in Equation (2):

(2) $$\log\;|G^{*}|=\delta +\frac{\alpha }{1+e^{\beta +\gamma \left({\log f}_{r}\right)}}$$

In the formula, $|G^{*}|$ is the complex modulus, in Pa; $f_{r}$ is the reduced frequency at the reference temperature, in rad/s; and δ, α, β, and γ are the fitting coefficients.

The calculation method of the master curve of the phase angle was consistent with that of the master curve of the complex modulus [33].

## 3. Results and Discussion

### 3.1. Influence of the Content of Mineral Powder on SBS Modified Asphalt Mastic

#### 3.1.1. Complex Modulus

**Figure 2:**
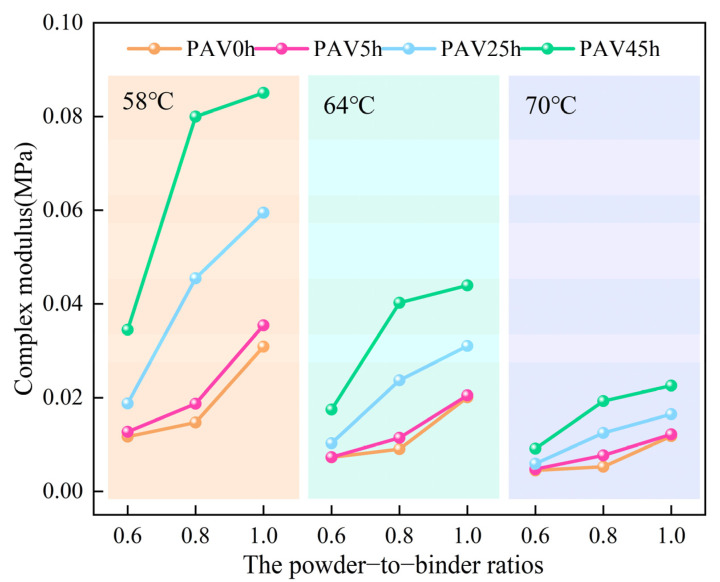
The complex modulus of SBS modified asphalt mastic samples with different powder-to-binder ratios under different temperatures and different degrees of aging.

The complex modulus reflects the stiffness of the asphalt material and can characterize the ability of the asphalt material to resist deformation under the action of repeated shear stress. It can be seen from Figure 2 that as the temperature increased, the complex modulus of the SBS modified asphalt mastic samples with various mineral powder contents decreased. This is because under high-temperature conditions, the molecular movement inside the mastic is intense and its ability to resist deformation deteriorates. As the temperature rose, the decrease in the complex modulus of the various mastic samples was the most significant. In practice, UV radiation also leads to an increase in the complex modulus due to continuous exposure to sunlight [34]. From the perspective of a single temperature, the complex modulus of the virgin SBS modified asphalt mastic gradually increased with increasing content of mineral powder. This indicated that a relatively high content of mineral powder could significantly enhance the deformation resistance of mastic under load. After aging in the PAV for 5 h (PAV5h), the complex modulus of the various mastic samples increased slightly. After the aging degree increased from PAV5h to PAV45h, the increasing rate of the complex modulus became larger. This is because the light components (saturate, aromatic) in the base asphalt volatilize and the hard components (asphaltene, resin) increase, which enhances the rigidity of the system. Moreover, as the content of mineral powder increased, the complex modulus of the mastic increased significantly at each level of aging, and the degree of hardening of the mastic became higher. Quantitative analysis revealed that, compared to the virgin SBS modified asphalt mastic, the complex modulus of SBSMA-0.6-PAV45h, SBSMA-0.8-PAV45h, and SBSMA-1.0-PAV45h at 58 °C exhibited respective increases of 195%, 444%, and 175%. This indicated that, during the aging process, the increase in the hardness of mastic does not show a simple positive correlation with the content of mineral powder. It is hypothesized that the factors governing the mastic aging process are not solely dependent on the mineral powder content but also involve complex interactions between SBS and the base asphalt matrix. To gain deeper insights into the factors governing the mastic aging process, the subsequent analysis will focus on the results of the phase angle measurements.

#### 3.1.2. Phase Angle

**Figure 3:**
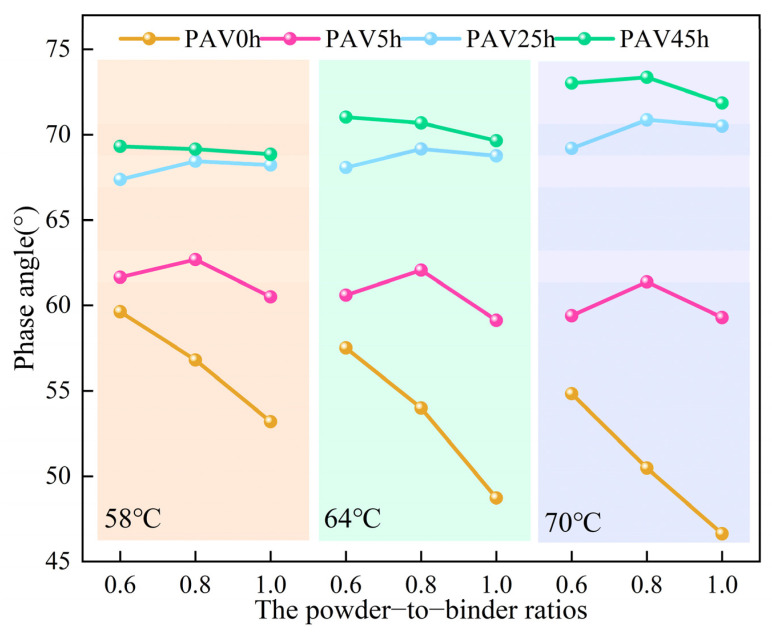
The phase angles of SBS modified asphalt mastic samples with different powder-to-binder ratios under different temperatures and different degrees of aging.

The phase angle represents the time lag between peak shear strain and peak shear stress, thereby reflecting the viscoelasticity of asphalt material to some degree [35]. The phase angle of SBS modified asphalt mastic often exhibits more complex viscoelastic characteristics, and the changes in the phase angle can reflect the state in which SBS exists. As can be seen from Figure 3, in the virgin state, the phase angle of the SBS modified asphalt mastic samples with each content of mineral powder decreased as the temperature increased, and the corresponding elastic component increased. This was contrary to the commonly held conclusion that the viscosity of asphalt increases with increasing temperature. This is because the SBS in SBS modified asphalt mastic is in an active state when the temperature rises. The double bonds of polybutadiene (PB) in SBS enhance the elasticity of asphalt, inhibiting the increase in asphalt viscosity caused by the rise in temperature. When the temperature increased, the phase angle of the mastic after PAV25h remained stable, indicating that the increase in elasticity caused by the activation of SBS counteracted the effect of the increase in viscosity caused by the softening of the base asphalt. When compared with the virgin SBS modified asphalt mastic, it was found that short-term aging led to the degradation of SBS, weakening the elastic effect of SBS. The phase angles of the SBS modified asphalt mastic aged for 25 h and 45 h in the PAV increased with the rise in temperature. This indicated that, at this time, the elastic enhancement effect of SBS was weaker than the softening effect of the base asphalt. After the superposition of these two effects, the result was an increase in viscosity. From these results, it can be seen that interactions between SBS and the base asphalt affect the properties of mastic.

From a specific temperature perspective, the mineral powder content exerts a significant influence on the aging behavior of asphalt mastic. In the virgin state, the phase angle of the mastic decreased as the content of mineral powder increased, indicating a positive correlation between the elasticity of mastic and the content of mineral powder. After PAV45h, the phase angle of the mastic with a relatively high content of mineral powder was still small, which was consistent with the variation pattern of the virgin mastic. This proved that mineral powder enhances the elasticity of mastic. From the perspective of the aging process, the phase angles of the mastic samples with powder-to-binder ratios of 0.6, 0.8, and 1.0 increased by 16%, 22%, and 29%, respectively, after PAV45h compared with those of the virgin mastic. Therefore, it could be seen that the variation range of the phase angle increased with increasing content of mineral powder, and a relatively high content of mineral powder could not effectively inhibit the softening of asphalt. These results preliminarily suggested that interactions occur between the mineral powder and SBS. A relatively high content of mineral powder accelerated the degradation of SBS and led to the loss of elasticity. Moreover, the effect of elastic loss caused by the degradation of SBS exceeded the effect of the elastic increase caused by the mineral powder. Under their comprehensive influence, the largest increase in the phase angle was brought about by a relatively high content of mineral powder.

The results of the phase angle and the complex modulus suggest that there is an interactive effect among the mineral powder, SBS, and the base asphalt, which jointly influence the viscoelasticity of mastic. Mineral powder can enhance the elastic effect of SBS modified asphalt mastic, but it will accelerate the loss of the elastic effect during the aging process. The aging of the base asphalt increases the hardness of mastic but fails to enhance its elasticity. SBS improves the elasticity of asphalt, but the degradation that occurs during the aging process causes the loss of elasticity. In other words, during the aging process, mineral powder promotes the degradation of SBS. The remaining base asphalt hardens sharply under the effect of pressure aging, thus resulting in an increase in the complex modulus. However, after PAV45h, the SBS was almost completely degraded, and at this time, the mastic could be regarded as a mixture composed of mineral powder and base asphalt. The mineral powder substituted for part of the elastic function of SBS, exhibiting a variation pattern wherein the phase angle increased as the content of mineral powder increased. Nevertheless, notable distinctions existed in the roles of the mineral powder between the virgin mastic and the mastic after PAV45h. In the virgin mastic, the mineral powder enhanced the elasticity of SBS modified asphalt mastic, while in the mastic after PAV45h, the mineral powder enhanced the elasticity of the base asphalt mastic.

#### 3.1.3. Recovery Rate

**Figure 4:**
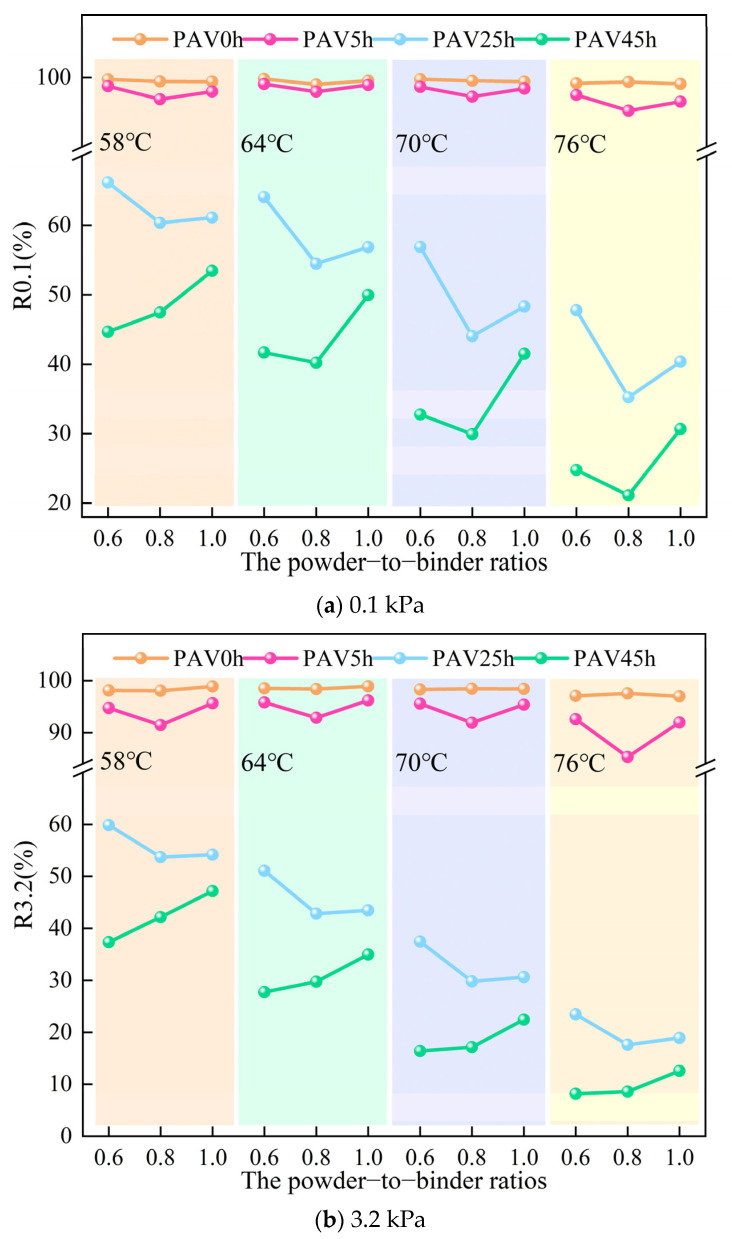
The R values of SBS modified asphalt mastic samples with different powder-to-binder ratios under different temperatures and degrees of aging.

The recovery rate (R) index is the result of the MSCR test conducted using a DSR. It can be used to measure the elastic deformation recovery ability of asphalt material. The larger the value of R, the better the asphalt mastic can return to its virgin state after deformation. The R value can further reflect the changes in the elastic effect of mastic based on the results of the phase angle in the temperature sweep test. Figure 4 shows the R values for the SBS modified asphalt mastic samples with different powder-to-binder ratios under different stress states, temperatures, and degrees of aging. When comparing the stress levels of 0.1 kPa and 3.2 kPa, an increase in stress led to a decrease in the deformation recovery rate, but it did not significantly change the main pattern of the recovery rate. Thus, the mastic R value at a stress level of 3.2 kPa will be described below. As shown in Figure 4b, with the increase in the test temperature, the value of R for the various mastic samples decreased. An increase in temperature led to a weakness in the elastic deformation recovery ability of SBS modified asphalt mastic.

As the degree of aging increased, this recovery ability decreased sharply, indicating that the aging process damages the temperature stability of mastic. Analyzing from the perspective of a single temperature, the R value of the virgin mastic increased with increasing content of mineral powder. At this time, the content of mineral powder affected the elastic recovery ability of the mastic, and this influence was positive. After PAV45h, the R value of the mastic still increased with increasing content of mineral powder, indicating that mineral powder can improve the elastic recovery properties of mastic. These phenomena were consistent with the results of the phase angle, suggesting that mineral powder itself has the ability to enhance the elastic effect of mastic, and this ability increases as the content of mineral powder increases. Similarly, the R value of the virgin mastic was jointly provided by the mineral powder, SBS, and the base asphalt. However, the R value after PAV45h was only provided by the mineral powder and the base asphalt. After PAV45h and PAV25h, the mastic samples with three different contents of mineral powder exhibited a very complex pattern of change, which was the result of the combined effects of SBS, the mineral powder, and the base asphalt.

#### 3.1.4. Irrecoverable Creep Flexibility

**Figure 5:**
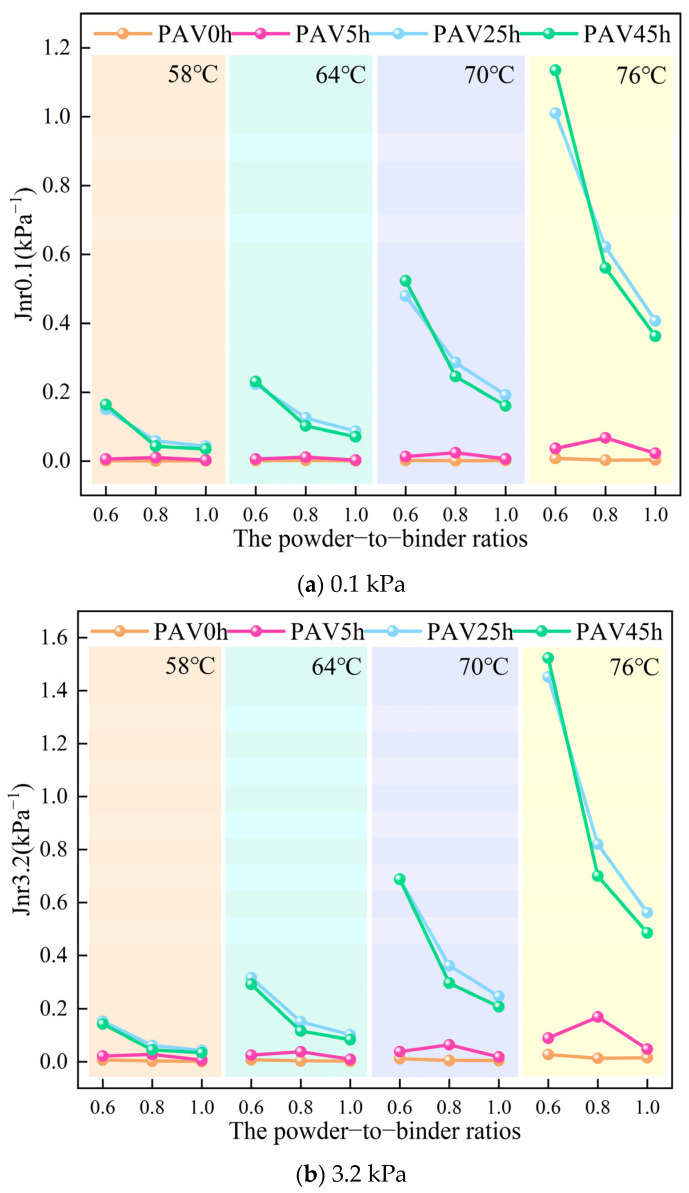
The Jnr values of SBS modified asphalt mastic samples with different powder-to-binder ratios under different temperatures and degrees of aging.

The irrecoverable creep flexibility (Jnr) is used to measure the residual deformation of asphalt [36]. The smaller the value of Jnr, the smaller the residual deformation of asphalt under external force will be, and the stronger the high-temperature deformation resistance will be. Figure 5 shows the Jnr0.1 and Jnr3.2 values of the SBS modified asphalt mastic samples with different powder-to-binder ratios under different temperatures and degrees of aging. Similarly, under stress levels of 0.1 kPa and 3.2 kPa, there was no obvious regular change in the Jnr values of the various asphalt mastic samples. Therefore, the following analysis is carried out under the stress level of 3.2 kPa. As depicted in Figure 5b, the virgin SBS-modified asphalt mastic demonstrated excellent high-temperature deformation resistance across diverse temperatures. With the progress of aging, the Jnr values of the various asphalt mastic samples increased, and the corresponding deformation resistance deteriorated. Moreover, as the temperature increased, the extent of this change further increased. The aging process damaged the high-temperature resistance of the asphalt mastic, resulting in poor high-temperature rutting resistance of the aged asphalt mastic. From the perspective of a single temperature, the Jnr values of the asphalt mastic samples with three different mineral powder contents all increased significantly after aging. Nevertheless, as the proportion of mineral powder rose, the magnitude of the increase in the Jnr value resulting from aging gradually declined. This finding suggested that mineral powder has the capability to impede the deformation of asphalt mastic induced by aging and enhance the rutting resistance of the aged asphalt mastic. Moreover, this improvement was positively correlated with the content of mineral powder. It is worth noting that the Jnr values of the various asphalt mastic samples after PAV25h and PAV45h were relatively close. There was even a situation where the Jnr values of the asphalt mastic samples with mineral powder contents of 0.8 and 1.0 after PAV45h were smaller than those after PAV25h. According to the above analysis results, the aging process of the asphalt mastic is jointly affected by the mineral powder, SBS and the base asphalt. And mineral powder may accelerate the degradation of SBS. This result led to the situation that, after PAV25h, a certain amount of SBS remained in the asphalt mastic sample with a mineral powder content of 0.6, and the deformation amount was naturally smaller than that after PAV45h. The asphalt mastic samples with mineral powder contents of 0.8 and 1.0 were greatly influenced by the mineral powder, and the degradation degree of SBS was relatively high when aged by PAV for 25 h. After PAV45h, the further hardening of the base asphalt improved the deformation resistance of the asphalt mastic, so the Jnr value at this time was lower than that after PAV25h again. Overall, the aging process of the asphalt mastic was indeed jointly affected by interactions among SBS, the mineral powder, and the base asphalt, and the effect of this influence was quite complex. In order to verify whether mineral powder has an impact on SBS, this paper will proceed with the frequency scanning study.

#### 3.1.5. Master Curve of Frequency Sweep

The master curve is a commonly used method for studying the rheological behavior of asphalt material [37,38]. According to the time–temperature equivalence principle, the results under low loading frequencies and high loading frequencies are respectively homologous to those under high temperatures and low temperatures [39,40]. In this paper, based on the results of the frequency sweep test, the rheological master curves of the complex modulus and phase angle were constructed for the SBS modified asphalt mastic with a powder-to-binder ratio of 0.6 and different degrees of aging, as well as for the SBS modified asphalt mastic with different powder-to-binder ratios after PAV25h. It was employed to characterize the impacts of the aging degree and the powder-to-binder ratio on the rheological properties of SBS modified asphalt mastic.

**Figure 6:**
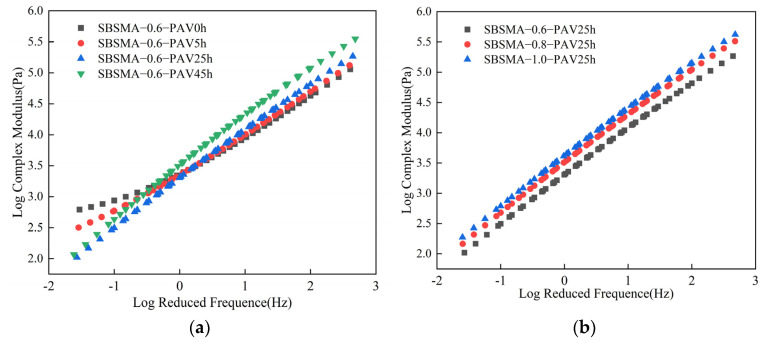
The master curves of the complex modulus of SBS modified asphalt mastic samples with different powder-to-binder ratios under different conditions: (**a**) Different degrees of aging; (**b**) Different powder-to-binder ratios.

As shown in Figure 6a, the asphalt mastic with a powder-to-binder ratio of 0.6 exhibited significant differences in the master curves of the complex modulus under different degrees of aging. In the low-frequency region, the master curve of the complex modulus of the unaged asphalt mastic did not show a strictly linear decrease as the frequency decreased. Instead, it intersected with the master curve of the aged asphalt mastic. This is because the SBS in the virgin asphalt mastic showed high activity at low frequencies, which inhibited the softening of the asphalt. As aging progressed, the SBS degraded, and this linear bending gradually decreased and disappeared. In the medium- and high-frequency domains, the pattern of variation exhibited by the master curve of the complex modulus bore a resemblance to the outcomes of the temperature sweep. The aging effect increased the hardness of the asphalt mastic, causing the complex modulus to increase. When the aging duration was controlled to be 25 h uniformly, the master curves of the complex modulus of the asphalt mastic samples under different powder-to-binder ratios were plotted, as shown in Figure 6b. The master curve of the complex modulus after PAV25h hardly showed the bending phenomenon in the low-frequency region, indicating that the SBS had almost completely degraded and lost its elastic effect. At this time, as the content of mineral powder increased, the master curve of the complex modulus moved upward, meaning that mineral powder can increase the hardness of asphalt mastic. The higher the content of mineral powder, the stronger the shear resistance of the asphalt mastic will be.

**Figure 7:**
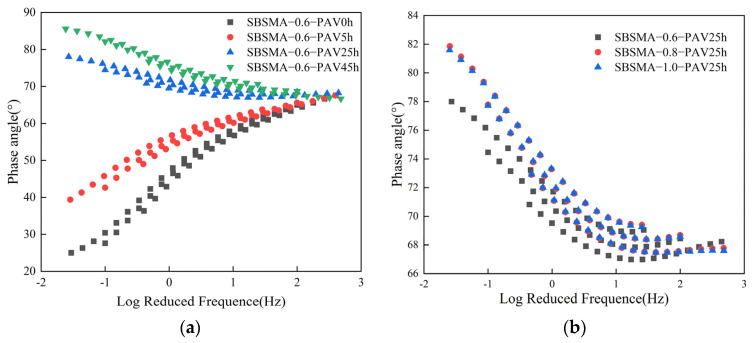
The master curves of the phase angle of SBS modified asphalt mastic samples with different powder-to-binder ratios under different conditions: (**a**) Different degrees of aging; (**b**) Different powder-to-binder ratios.

The same method was used to analyze the variation pattern of the master curve of the phase angle. The master curve of the phase angle can reflect the integrity of SBS to a certain extent. The powder-to-binder ratio was controlled to be 0.6 uniformly, and the influence of different aging durations on the asphalt mastic was studied, as shown in Figure 7a. As the frequency increased, the master curve of the phase angle of the virgin asphalt mastic gradually increased. The master curve of the phase angle of the asphalt mastic shifted upward after aging. This indicated that the aging process led to the degradation of SBS, suppressing the elastic effect of the asphalt mastic. The aging duration was controlled to be 25 h, and the content of mineral powder was changed, as shown in Figure 7b. The master curves with a powder-to-binder ratio of 0.8 and 1.0 were significantly higher than that with a powder-to-binder ratio of 0.6. Moreover, the master curve with a powder-to-binder ratio of 1.0 was higher than that with a ratio of 0.8 at a relatively large number of frequencies. This indicated that the content of mineral powder has an impact on the degradation of SBS, and as the content of mineral powder increases, the degradation rate of SBS accelerates.

### 3.2. Influence of Mineral Powder Gradation on SBS Modified Asphalt Mastic

#### 3.2.1. Complex Modulus

In the above text, mineral powder with a particle size of 0.075 was used. To conduct a more profound investigation into the impact of diverse fineness levels of mineral powder on asphalt mastic, a 500 mesh mineral powder sieve was employed to perform the sieving test. As a result, two gradations of mineral powder were differentiated. The same content of the two gradations of mineral powder was respectively added to SBS modified asphalt to prepare the asphalt mastic, and then the aging test was carried out.

**Figure 8:**
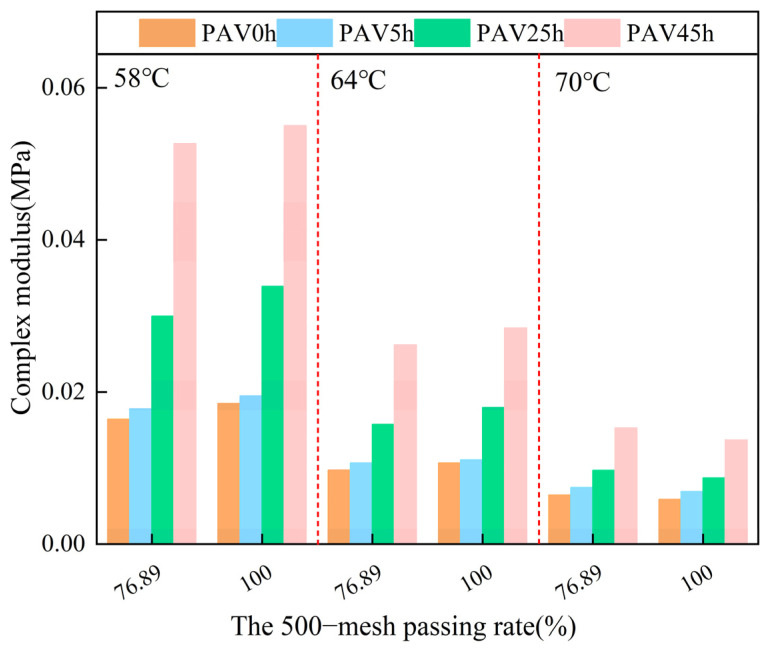
The complex modulus of SBS modified asphalt mastic samples with different mineral powder gradations under different conditions.

The complex modulus of the asphalt mastic samples with different mineral powder gradations was obtained through the temperature sweep test, as shown in Figure 8. When the temperature rose, the complex modulus of the various asphalt mastic samples decreased significantly, and the hardness of the asphalt mastic dropped. When considering the single temperature condition, as the particle size of the mineral powder decreased, the corresponding complex modulus of the asphalt mastic increased. The underlying reason lies in the fact that with a finer gradation of the mineral powder, the specific surface area of its particles becomes larger. As a result, the contact area between the mineral powder and the asphalt is increased, and the interaction ability between the asphalt and the mineral powder is improved, thus enhancing the deformation resistance of the system. As the degree of aging deepened, the complex modulus of the asphalt mastic gradually increased, which was similar to the conclusion in the previous text.

#### 3.2.2. Phase Angle

**Figure 9:**
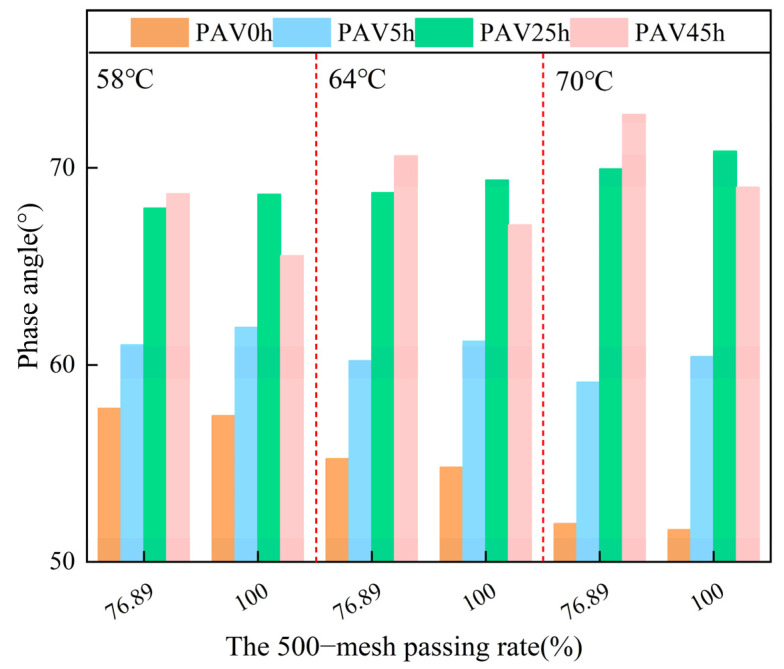
The phase angle of SBS modified asphalt mastic samples with different mineral powder gradations under different conditions.

In order to more clearly observe the influence of the aging process on SBS modified asphalt mastic, it was also necessary to conduct an analysis of the viscoelastic changes of aged asphalt mastic. As shown in Figure 9, the phase angle of the virgin asphalt mastic was less affected by the particle size of the mineral powder. At this time, the SBS provided the main properties of the asphalt mastic. As the temperature increased, the phase angles of the two virgin asphalt mastic samples decreased, which was similar to the previous results. During the process of temperature increase, the phase angles of the two aged asphalt mastic samples gradually increased, indicating that the SBS in the asphalt mastic degraded at this time, and the elasticity of the asphalt mastic decreased. After PAV5h and PAV25h, the phase angle of the fine-gradation asphalt mastic was slightly higher than that of the coarse-gradation asphalt mastic, indicating that the mineral powder with a smaller particle size was more likely to accelerate the degradation of SBS, resulting in an increase in the overall viscosity of the asphalt mastic. However, after PAV45h, the phase angle of the fine-graded asphalt mastic was significantly lower than that of the mastic with large particle sizes, exhibiting a remarkable elastic effect. This indicated that the SBS had been completely degraded at this point. The mineral powder provided part of the elasticity for the asphalt mastic, and the mineral powder with smaller particle sizes offered a stronger elastic effect than that with larger particle sizes.

#### 3.2.3. Recovery Rate

**Figure 10:**
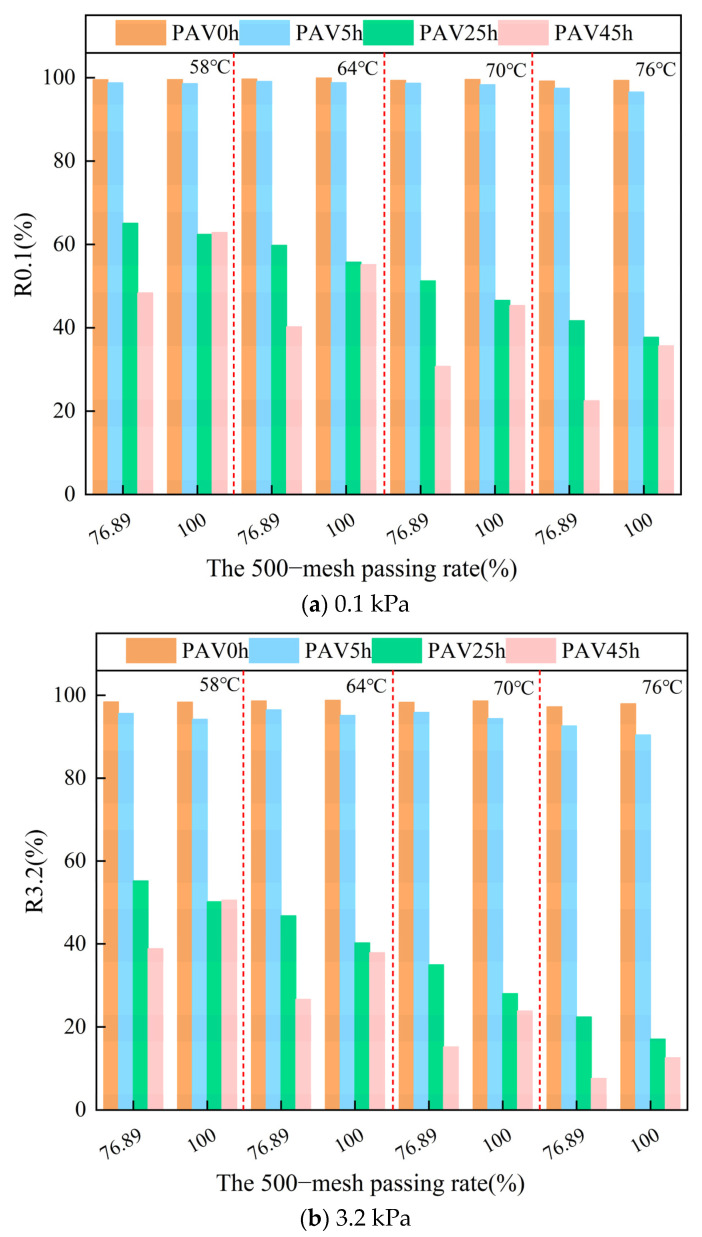
The R values of SBS modified asphalt mastic samples with different gradations under different conditions.

The MSCR test was continued, and the difference in the elastic capacity provided by mineral powders with different particle sizes was verified through the R value. As shown in Figure 10, the R values of the asphalt mastic samples under different stresses were similar. As shown in Figure 10b, after the temperature increased, the variation pattern of the R value of the asphalt mastic samples with different gradations was similar to the results of the phase angle, and it will not be elaborated here again. At the same temperature, due to the effect of SBS, the R value of the two virgin asphalt mastic samples always remained at a relatively high level. At this moment, the asphalt mastic samples were mainly influenced by SBS and exhibited a relatively high elastic recovery ability. After PAV5h, the R value of the fine-graded asphalt mastic was slightly lower than that of the coarse-graded asphalt mastic, indicating that the SBS started to degrade. After PAV25h, the R value of the fine-graded asphalt mastic was significantly lower than that of the coarse-graded asphalt mastic, suggesting that the SBS underwent a large degree of degradation. After PAV45h, a phenomenon similar to the results of the temperature sweep phase angle occurred. The R value of the fine-graded asphalt mastic was significantly greater than that of the coarse-graded asphalt mastic. This indicated that, at this time, the SBS had almost completely degraded, and the mineral powder provided elasticity for the aged asphalt mastic. Moreover, the smaller the particle size, the stronger the ability to provide elasticity, and the better the elastic recovery ability of the asphalt mastic.

#### 3.2.4. Irrecoverable Creep Flexibility

**Figure 11:**
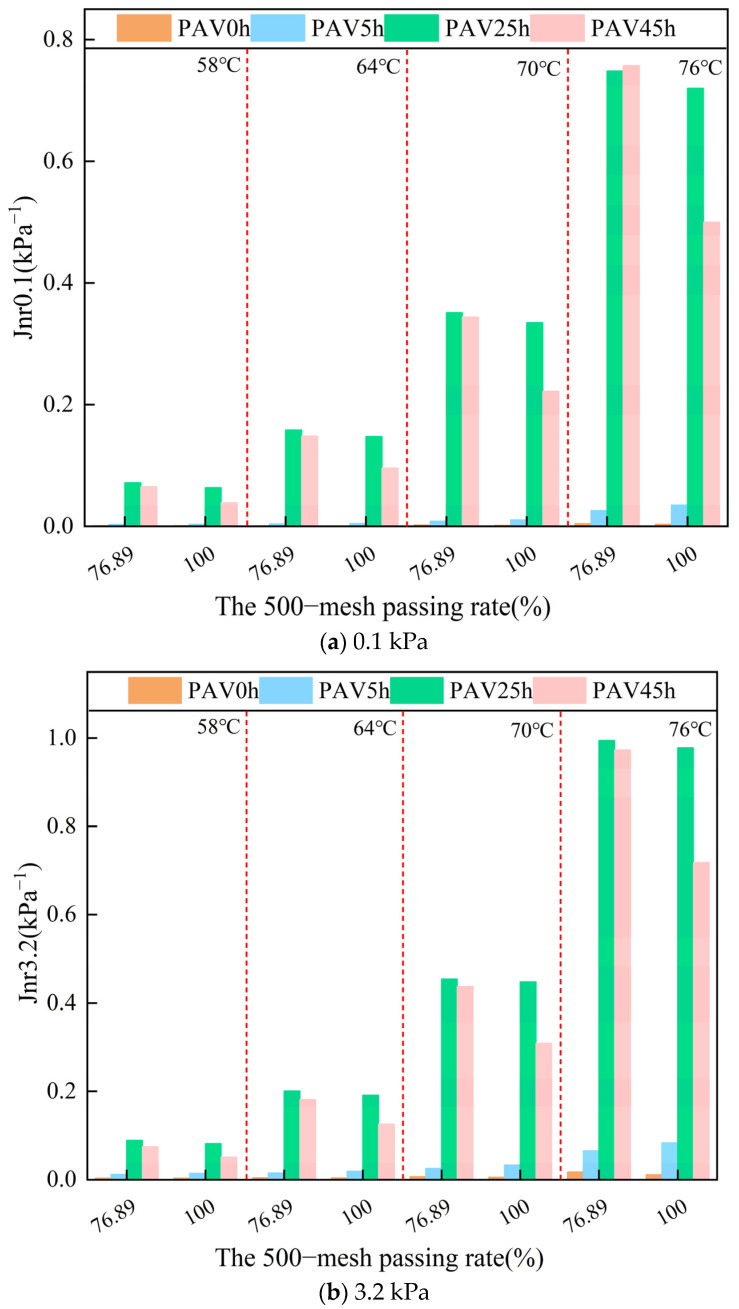
The Jnr values of SBS modified asphalt mastic samples with different gradations under different conditions.

The gradation of mineral powder will affect the Jnr value of SBS modified asphalt mastic. In Figure 11, with respect to the stress levels of 0.1 Kpa and 3.2 Kpa in both the unaged and aged states of the asphalt mastic, a more refined gradation of the mineral powder was associated with a reduction in the corresponding Jnr value. This indicated that the irreversible deformation of SBS modified asphalt mastic after being subjected to stress decreased, and the deformation resistance at high temperatures was improved. Moreover, after PAV45h, the reduction range of the Jnr value of the fine-graded asphalt mastic was larger than that of the coarse-graded asphalt mastic. This indicated that, under this aged condition, the deformation resistance of asphalt mastic can be improved by increasing the fineness of the mineral powder.

#### 3.2.5. Master Curve of Frequency Sweep

**Figure 12:**
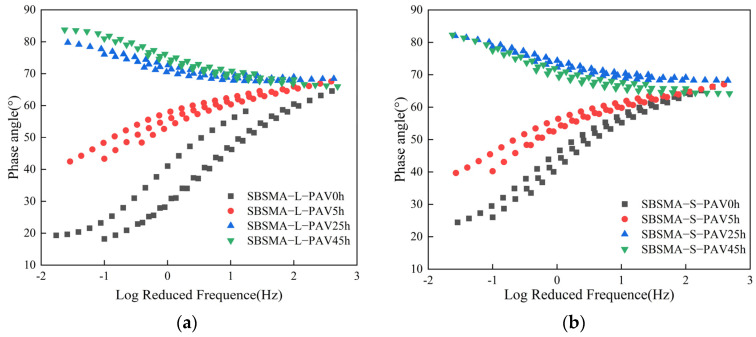
The master curves of the phase angles of SBS modified asphalt mastic samples with different gradations under different degrees of aging: (**a**) Passing rate of 76.89%; (**b**) Passing rate of 100%.

Next, the degradation of SBS in the asphalt mastic during different aging processes was observed through the master curve of the phase angle. Figure 12a and b respectively depict the master curves of the phase angles of the coarse-graded asphalt mastic and the fine-graded asphalt mastic under different degrees of aging. As the degree of aging deepened, the master curve of the phase angle gradually shifted upward in the low-frequency region, which was influenced by the degree of SBS degradation. In its virgin state, SBS was intact and provided elasticity to the asphalt mastic. The lower the frequency, the more active the PB segment in SBS will be, and the phase angle decreased as the frequency decreased. After aging, SBS degraded, and the master curve of the phase angle gradually shifted upward. In comparing the master curves of the phase angles of the two graded asphalt mastic samples, it can be seen that there are significant differences between them at PAV25h and PAV45h. During the process from PAV25h to PAV45h, the variation range of the master curve of the phase angle was very small, indicating that the SBS was almost completely degraded at this time. After the fine-graded asphalt mastic was aged for 45 h, the small particle-sized mineral powder inside can provide a stronger elastic effect. At this time, the only factor contributing to the elastic enhancement was the mineral powder with different particle sizes. The small particle-sized mineral powder provided greater elasticity, which in turn made the master curve of the phase angle lower than that at PAV25h. On the contrary, the elastic enhancement effect of the large particle-sized mineral powder was limited. The aging of the base asphalt exceeded the elastic enhancement effect of the large particle-sized mineral powder, and the interaction led to a further decrease in elasticity.

**Figure 13:**
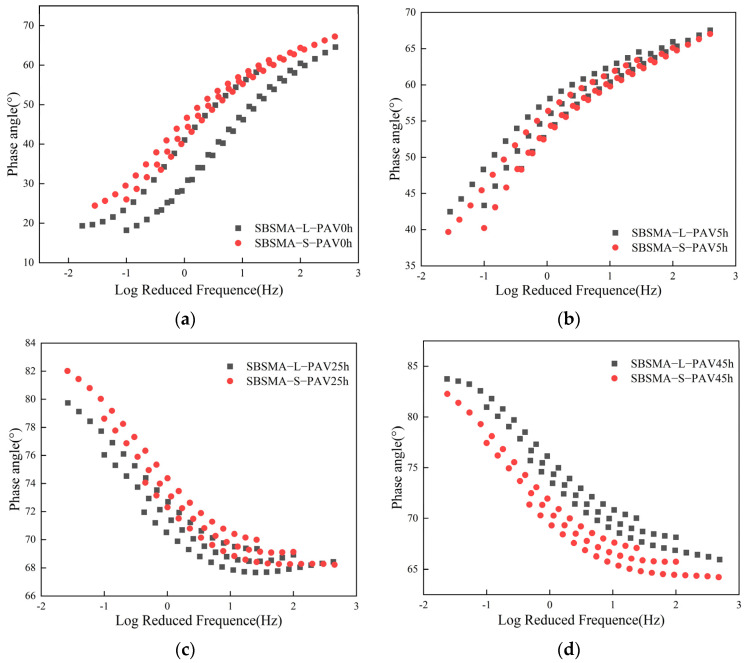
The master curves of the phase angles of SBS modified asphalt mastic samples with different gradations under four degrees of aging: (**a**) PAV0h; (**b**) PAV5h; (**c**) PAV25h; (**d**) PAV45h.

Figure 13 demonstrates the dynamic degradation of SBS in the two graded asphalt mastic samples during the aging process. During the aging process, the phase angle of the fine-graded asphalt mastic gradually moved downward from a relatively high value, gradually intersected with that of the coarse-graded asphalt mastic, and finally moved below the phase angle of coarse-graded asphalt mastic. Judging from the results, SBS gradually degraded during the aging process, and the fine-graded asphalt mastic exhibited a higher elastic effect than the coarse-graded asphalt mastic. As is known from the content in Section 3.1, mineral powder can replace part of the function of SBS and provide elasticity for the aged asphalt mastic.

## 4. Conclusions

In this paper, samples of SBS modified asphalt mastic with limestone mineral powder with powder-to-binder ratios of 0.6, 0.8, and 1.0 were respectively prepared, as well as samples of SBS modified asphalt mastic with limestone mineral powder having a powder-to-binder ratio of 0.8 and 500 mesh passing rates of 76.89% and 100%, respectively. And the above samples were subjected to thermal-oxidative aging for 5 h, 25 h, and 45 h through PAV. Then, temperature sweep, MSCR, and frequency sweep tests were carried out using a DSR to explore the influences of the content and gradation of the mineral powder on the high-temperature rheological properties of the asphalt mastic, and the following conclusions were drawn:

(1) Mineral powder improves the overall deformation resistance of asphalt mastic. As the content of mineral powder increases, the deformation resistance of asphalt mastic is enhanced. The mineral powder itself can provide elasticity for the asphalt mastic, and the elastic effect of the asphalt mastic increases with increasing content of mineral powder. A relatively high content of mineral powder can improve the long-term anti-aging ability of asphalt mastic.
(2) During the aging process, the viscoelastic properties of asphalt mastic are influenced by interactions among SBS, the mineral powder, and the base asphalt. During the aging process, a relatively high proportion of mineral powder can endow asphalt mastic with greater elasticity. However, when the content of mineral powder increases, the degradation rate of SBS accelerates. Coupled with the continuous hardening of the base asphalt, SBS modified asphalt mastic exhibits a high degree of complexity during the aging process.
(3) Mineral powder with smaller particle sizes can enhance the deformation resistance of asphalt mastic more effectively than that with larger particle sizes. The rutting resistance of the fine-graded asphalt mastic after 45 h of aging exceeded that after 25 h of aging, because the mineral powder compensated for the decrease in the elasticity of the asphalt mastic caused by the loss of SBS and provided strength for the asphalt mastic. A finer gradation corresponds to a higher strength being provided.
(4) The smaller the particle size of the mineral powder, the better the anti-aging properties of the corresponding fine-graded asphalt mastic. After 45 h of long-term aging, the mineral powder with smaller particle sizes provided a superior elastic effect to the asphalt mastic compared to that of the mineral powder with larger particle sizes. Although mineral powder can compensate for part of the degradation loss of SBS, there are significant differences between the roles of mineral powder and SBS in enhancing the elasticity of asphalt mastic.
(5) Due to the limited raw materials for the project, we only chose one type of SBS modified asphalt to prepare the corresponding mastic for the corresponding experimental investigation. Whether the results are applicable to other SBS formulations or different base asphalt compositions needs to be verified by further tests. Considering SEM or XRD to provide insights into how the mineral powder disperses and interacts with the binder, particularly at different aging stages, will be a priority in our follow-up research. In order to explore the effects of aging in realistic environments, we will consider the effects of oxidative, UV, and moisture-related aging in subsequent studies.

## Figures and Tables

**Table 1:** Basic properties of base asphalt and corresponding SBS modified asphalt.

Type	Base Asphalt	SBS Modified Asphalt
Penetration (25 °C)/0.1 mm	83	71
Softening Point (°C)	46	81
Ductility (5 °C, cm)	/	40.7
Ductility (15 °C, cm)	>100	/
Flash Point	/	>230

**Table 2:** The full names of the nouns in the pictures and their abbreviations with definitions.

Number	Terminology	Abbreviation
1	Powder-to-binder ratio	P/B
2	Pass rate	PR
3	SBS modified asphalt + P/B 0.6 filler	SBSMA-0.6-PAV0h
4	SBS modified asphalt + P/B 0.6 filler + PAV5h	SBSMA-0.6-PAV5h
5	SBS modified asphalt + P/B 0.6 filler + PAV25h	SBSMA-0.6-PAV25h
6	SBS modified asphalt + P/B 0.6 filler + PAV45h	SBSMA-0.6-PAV45h
7	SBS modified asphalt + P/B 0.8 filler	SBSMA-0.8-PAV0h
8	SBS modified asphalt + P/B 0.8 filler + PAV5h	SBSMA-0.8-PAV5h
9	SBS modified asphalt + P/B 0.8 filler + PAV25h	SBSMA-0.8-PAV25h
10	SBS modified asphalt + P/B 0.8 filler + PAV45h	SBSMA-0.8-PAV45h
11	SBS modified asphalt + P/B 1.0 filler	SBSMA-1.0-PAV0h
12	SBS modified asphalt + P/B 1.0 filler + PAV5h	SBSMA-1.0-PAV5h
13	SBS modified asphalt + P/B 1.0 filler + PAV25h	SBSMA-1.0-PAV25h
14	SBS modified asphalt + P/B 1.0 filler + PAV45h	SBSMA-1.0-PAV45h
15	SBS modified asphalt + pass rate 76.89% filler	SBSMA-L-PAV0h
16	SBS modified asphalt + pass rate 76.89% filler + PAV5h	SBSMA-L-PAV5h
17	SBS modified asphalt + pass rate 76.89% filler +PAV25h	SBSMA-L-PAV25h
18	SBS modified asphalt + pass rate 76.89% filler + PAV45h	SBSMA-L-PAV45h
19	SBS modified asphalt + pass rate 100% filler	SBSMA-S-PAV0h
20	SBS modified asphalt + pass rate 100% filler + PAV5h	SBSMA-S-PAV5h
21	SBS modified asphalt + pass rate 100% filler + PAV25h	SBSMA-S-PAV25h
22	SBS modified asphalt + pass rate 100% filler + PAV45h	SBSMA-S-PAV45h

## Data Availability

The original contributions presented in this study are included in the article. Further inquiries can be directed to the corresponding authors.
